# Supplementary material for: EnzML: multi-label prediction of enzyme classes using InterPro signatures
Source: BMC Bioinformatics. 2012 Apr 25;13:61. doi: 10.1186/1471-2105-13-61 (PMC3483700; doi:10.1186/1471-2105-13-61)
Supplement: Addtional file 5 — The Java code to format the data files, evaluate and predict. The file enzml_java_code.tar.gz contains the Java code used to format database data to ARFF and XML formats, to execute cross and train-test (jackknife) evaluations and to record evaluation results to database. More information is included in the readme.txt file and the Javadoc files. The code can be used with a MySQL database. To use a different database software, other JDBC drivers might be required. [file 1471-2105-13-61-S5.gz › java_code/utils/doc/uk/ac/ed/inf/utils/Utils.html]

Utils


---


|  |  |  |
| --- | --- | --- |
| **Overview** \| **Package** \| **Class** \| **Use** \| **Tree** \| **Deprecated** \| **Index** \| **Help** | |  |
| **PREV CLASS**   **NEXT CLASS** | **FRAMES**    **NO FRAMES**     **All Classes** |  |
| SUMMARY: NESTED \| FIELD \| CONSTR \| METHOD | DETAIL: FIELD \| CONSTR \| METHOD |  |


---


## uk.ac.ed.inf.utils Class Utils

```
java.lang.Object
  uk.ac.ed.inf.utils.Utils
```

---

``` public class Utils extends java.lang.Object ```

Various utilities to manipulate vectors of numbers, get a timestamp etc.

**Version:**
:   24-Mar-2007

**Author:**
:   Luna De Ferrari (luna.deferrari at ed.ac.uk)

---

**Constructor Summary**

`Utils()`


| **Method Summary** | |
| --- | --- |
| `static double` | `calcAverage(double[] values)`             Calculates the average of the numbers in an array (from web http://forum.java.sun.com/, author JosAH) |
| `static double` | `calcStandardDeviation(double[] values)`             Calculates the standard deviation of the elements in an array as root of the variance = [sum( x(i) - mean)^2 )] / (n-1), where x(i) is the ith element, m the mean, n the number of elements |
| `static java.util.TreeSet<java.lang.Double>` | `getDoubleIntervals(java.lang.Double min, java.lang.Double max, java.lang.Double percentOfIncrement)` |
| `static boolean` | `getFlag(java.lang.String opt, java.lang.String[] options)` |
| `static java.util.TreeSet` | `getIncrements(java.lang.Object min, java.lang.Object max, java.lang.Double percentageOfIncrement)`             Returns a vector of numbers, starting from a minimum value, with incremental increases until the maximum value. |
| `static java.util.TreeSet<java.lang.Integer>` | `getIncrementsFromRange(java.lang.Integer range, java.lang.Double percentageOfIncrement)`             Returns a set of increments. |
| `static java.util.TreeSet<java.lang.Integer>` | `getIntegerIntervals(java.lang.Integer min, java.lang.Integer max, java.lang.Double percentageOfIncrement)`             Returns a vector of numbers, starting from a minimum value, with incremental increases until the maximum value. |
| `static void` | `getJavaSystemProperties()`             Gets the Java system properties |
| `static java.lang.String` | `getOption(java.lang.String opt, java.lang.String[] options)`             getOption(String opt, String[] options) gets an option value out of a list of options |
| `static java.util.TreeSet<java.lang.String>` | `getSimpleSerie(java.lang.Double min, java.lang.Double increment, int iterations)`             Gets a series of doubles from a min incrementing by a given increment for a number of iterations |
| `static void` | `main(java.lang.String[] argv)`             Main method for testing |
| `static java.util.Properties` | `readLocalProperties(java.lang.String resourceName)`             Reads properties that inherit from three locations. |
| `static java.util.Properties` | `readProperties(java.lang.String propertiesFilePath)`             Reads properties Code adapted from Weka: http://www.cs.waikato.ac.nz/ml/weka/ Ian H. |
| `static double` | `roundDouble(double targetDouble, int decimalPlace)`             Rounds a number to just n decimal digits |
| `static java.util.TreeSet<java.lang.String>` | `setFromObjectsToString(java.util.TreeSet<java.lang.Object> set)` |

**Methods inherited from class java.lang.Object**

`equals, getClass, hashCode, notify, notifyAll, toString, wait, wait, wait`

**Constructor Detail**

### Utils

```
public Utils()
```


**Method Detail**

### calcAverage

```
public static double calcAverage(double[] values)
```

:   Calculates the average of the numbers in an array (from web
    http://forum.java.sun.com/, author JosAH)

    :   **Returns:**: average of the numbers (rounded to 2 decimal digits)

---


### calcStandardDeviation

```
public static double calcStandardDeviation(double[] values)
```

:   Calculates the standard deviation of the elements in an array as root of
    the variance = [sum( x(i) - mean)^2 )] / (n-1), where x(i) is the ith
    element, m the mean, n the number of elements

    :   **Returns:**: standard deviation

---


### getDoubleIntervals

```
public static java.util.TreeSet<java.lang.Double> getDoubleIntervals(java.lang.Double min,
                                                                     java.lang.Double max,
                                                                     java.lang.Double percentOfIncrement)
```

---


### getFlag

```
public static boolean getFlag(java.lang.String opt,
                              java.lang.String[] options)
```

---


### getIncrements

```
public static java.util.TreeSet getIncrements(java.lang.Object min,
                                              java.lang.Object max,
                                              java.lang.Double percentageOfIncrement)
```

:   Returns a vector of numbers, starting from a minimum value, with
    incremental increases until the maximum value. Example: for min 2 and max
    12, percentage of increment 0.2 > [2, 4, 6, 8, 10, 12]

    :   **Parameters:**: `min` - the minimum number value: `max` - the maximum number value: `percentageOfIncrement` - the increment as percentage of (maximum - minimum) value (the actual increment value has to be >= 1 !) **Returns:**: a vector of numbers, starting from a minimum value, with incremental increases until the maximum value. Returns null if the increment is 0.

---


### getIncrementsFromRange

```
public static java.util.TreeSet<java.lang.Integer> getIncrementsFromRange(java.lang.Integer range,
                                                                          java.lang.Double percentageOfIncrement)
```

:   Returns a set of increments. Example: for range 10, percentage of
    increment 0.2 > [0, 2, 4, 6, 8, 10]

    :   **Parameters:**: `range` -: `percentageOfIncrement` - **Returns:**: a set of increments

---


### getIntegerIntervals

```
public static java.util.TreeSet<java.lang.Integer> getIntegerIntervals(java.lang.Integer min,
                                                                       java.lang.Integer max,
                                                                       java.lang.Double percentageOfIncrement)
```

:   Returns a vector of numbers, starting from a minimum value, with
    incremental increases until the maximum value.

    :   **Parameters:**: `min` - the minimum number value: `max` - the maximum number value: `percentageOfIncrement` - the increment as percentage of (maximum - minimum) value **Returns:**: a vector of numbers, starting from a minimum value, with incremental increases until the maximum value. Returns null if the increment is 0.

---


### getJavaSystemProperties

```
public static void getJavaSystemProperties()
```

:   Gets the Java system properties

---


### getOption

```
public static java.lang.String getOption(java.lang.String opt,
                                         java.lang.String[] options)
```

:   getOption(String opt, String[] options) gets an option value out of a
    list of options

    :   **Parameters:**: `opt` - the option that needs to be extracted (e.g. -x): `options` - list of options (e.g. -x 10 -y name) **Returns:**: the value for the option selected (e.g. 10)

---


### getSimpleSerie

```
public static java.util.TreeSet<java.lang.String> getSimpleSerie(java.lang.Double min,
                                                                 java.lang.Double increment,
                                                                 int iterations)
```

:   Gets a series of doubles from a min incrementing by a given increment for
    a number of iterations

    :   **Parameters:**: `min` - the starting point: `increment` - the increment: `iterations` - the number of repetitions **Returns:**: a series of doubles from a min incrementing by a given increment for a number of iterations

---


### main

```
public static void main(java.lang.String[] argv)
                 throws java.lang.Exception
```

:   Main method for testing

    :   **Parameters:**: `argv` - **Throws:**: `java.lang.Exception`

---


### readLocalProperties

```
public static java.util.Properties readLocalProperties(java.lang.String resourceName)
                                                throws java.lang.Exception
```

:   Reads properties that inherit from three locations. Code from Weka:
    http://www.cs.waikato.ac.nz/ml/weka/
    Ian H. Witten and Eibe Frank (2005)
    "Data Mining: Practical machine learning tools and techniques", 2nd
    Edition, Morgan Kaufmann, San Francisco, 2005.
    Reads properties that inherit from three locations. Properties are first
    defined in the system resource location (i.e. in the CLASSPATH). These
    default properties must exist. Properties defined in the users home
    directory (optional) override default settings. Properties defined in the
    current directory (optional) override all these settings.

    :   **Parameters:**: `resourceName` - the location of the resource that should be loaded. e.g.: "weka/core/Utils.props". (The use of hardcoded forward slashes here is OK - see jdk1.1/docs/guide/misc/resources.html) This routine will also look for the file (in this case) "Utils.props" in the users home directory and the current directory. **Returns:**: the Properties **Throws:**: `java.lang.Exception` - if no default properties are defined, or if an error occurs reading the properties files.

---


### readProperties

```
public static java.util.Properties readProperties(java.lang.String propertiesFilePath)
                                           throws java.lang.Exception
```

:   Reads properties Code adapted from Weka:
    http://www.cs.waikato.ac.nz/ml/weka/
    Ian H. Witten and Eibe Frank (2005)
    "Data Mining: Practical machine learning tools and techniques", 2nd
    Edition, Morgan Kaufmann, San Francisco, 2005.

    :   **Returns:**: the Properties **Throws:**: `java.lang.Exception` - if no default properties are defined, or if an error occurs reading the properties files.

---


### roundDouble

```
public static double roundDouble(double targetDouble,
                                 int decimalPlace)
```

:   Rounds a number to just n decimal digits

    :   **Parameters:**: `targetDouble` - the number to be rounded: `decimalPlace` - the number of digits to be left right of the decimal dot **Returns:**: the rounded number

---


### setFromObjectsToString

```
public static java.util.TreeSet<java.lang.String> setFromObjectsToString(java.util.TreeSet<java.lang.Object> set)
```


---


|  |  |  |
| --- | --- | --- |
| **Overview** \| **Package** \| **Class** \| **Use** \| **Tree** \| **Deprecated** \| **Index** \| **Help** | |  |
| **PREV CLASS**   **NEXT CLASS** | **FRAMES**    **NO FRAMES**     **All Classes** |  |
| SUMMARY: NESTED \| FIELD \| CONSTR \| METHOD | DETAIL: FIELD \| CONSTR \| METHOD |  |


---
